# Best Practices for the Implementation and Sustainment of Virtual Health Information System Training: Qualitative Study

**DOI:** 10.2196/30613

**Published:** 2021-10-22

**Authors:** Tharshini Jeyakumar, Sharon Ambata-Villanueva, Sarah McClure, Carolyn Henderson, David Wiljer

**Affiliations:** 1 University Health Network Toronto, ON Canada; 2 Institute of Health Policy, Management and Evaluation Dalla Lana School of Public Health University of Toronto Toronto, ON Canada; 3 University of Toronto, Faculty of Medicine Toronto, ON Canada; 4 CAMH Education Centre for Addictions and Mental Health Toronto, ON Canada

**Keywords:** training, health care providers, educational technology, patient care, COVID-19, development, practice, best practice, pedagogy, teaching, implementation, medical education, online education, care delivery, perception, effectiveness

## Abstract

**Background:**

The COVID-19 pandemic has necessitated the adoption and implementation of digital technologies to help transform the educational ecosystem and the delivery of care.

**Objective:**

We sought to understand instructors’ and learners’ perceptions of the challenges and opportunities faced in implementing health information system virtual training amid the COVID-19 pandemic.

**Methods:**

Semistructured interviews were conducted with education specialists and health care staff who provided or had taken part in a virtual instructor-led training at a large Canadian academic health sciences center. Guided by the Technology Acceptance Model and the Community of Inquiry framework, we analyzed interview transcript themes deductively and inductively.

**Results:**

Of the 18 individuals participating in the study, 9 were education specialists, 5 were learners, 3 were program coordinators, and 1 was a senior manager at the Centre for Learning, Innovation, and Simulation. We found 3 predominant themes: adopting a learner-centered approach for a meaningful learning experience, embracing the advances in educational technologies to maximize the transfer of learning, and enhancing the virtual user experience.

**Conclusions:**

This study adds to the literature on designing and implementing virtual training in health care organizations by highlighting the importance of recognizing learners’ needs and maximizing the transfer of learning. Findings from this study can be used to help inform the design and development of training strategies to support learners across an organization during the current climate and to ensure changes are sustainable.

## Introduction

COVID-19 has created social disruptions and contributed to unprecedented changes that have required that many organizations rapidly transition instructor-led classes to a virtual format [1]. The emergence and innovation of digital technology have played important roles in education and training. Virtual education and training for health information systems are critical in supporting timely care and patient safety and in preventing information technology–related patient harm [2]. Virtual instructor-led training has been defined as web-based education delivered via digital platforms, connecting instructors and learners across different geographic boundaries [3]. Education refers to the acquisition of knowledge and skills, enabling learners to develop reasoning and judgement surrounding broad topics [4,5]. Conversely, training is the process of teaching the skills that one needs to perform a particular job or activity [4], which is the emphasis of this study. Virtual training environments are designed to simulate classroom instruction and can be conducted synchronously or asynchronously [3]. Virtual instructor-led training is poised to transform health care across the country as organizations shift toward rapidly providing training to health care providers and staff, leading to a paradigm shift that could reshape the provision of training long-term (ie, in postpandemic conditions) [6].

Although virtual training has been an emergent part of health professional education for over a decade, this abrupt pivot to teaching virtually has become a significant challenge [7]. Challenges exist with the implementation of the change process in the education system due to the novel perspectives of virtual training and its technological complexities. Stakeholders may not be prepared to adjust to this shift to virtual training, as they are not technologically ready to adapt to this postpandemic educational landscape [8]. Moreover, Ali [9] asserted that adopting a virtual training environment is not only a technical issue but also a pedagogical and instructional challenge. With the focus on digital and technology-enabled learning, it is imperative to understand the required elements of virtual training and how the existing resources of institutions can be leveraged to effectively transform face-to-face instruction into virtual instruction [8].

A recent paper [2] discussed the accelerated development and implementation of a blended electronic medical record learning strategy for nursing staff across a large urban health care district in Sydney, Australia; the study demonstrated that virtual training’s accessibility and flexibility provided a practical approach for delivering electronic medical record training to nursing staff, particularly in the context of an urgent need [2]. Collaboration with stakeholders was identified as a key feature in facilitating the rapid conversion of face-to-face training into a virtual approach. The feedback gathered from participants were self-reported measures, and thus, may not be reflective of practice observed in clinical settings [2].

Virtual delivery provides a promising avenue for offering an optimal learning experience while accommodating the various needs of learners due to geographic, physical, or other limitations [10]. Even though studies have reported that virtual training can be as effective as traditional instructor-based sessions, it is imperative to consider factors such as having clearly defined guidelines about the instructor’s roles and responsibilities, learner competencies, technology accessibility, and funding [7]. Mishra et al [8] stated that theatrical skills are needed to successfully integrate technology into the teaching-learning process. Theatrical skills include empathy, mindfulness of learner needs, strong presentation skills, and the appropriate use of digital technologies to transmit some level of embodied experience and knowledge [8]. Virtual training is not only an advantageous step toward the future of education, but it is also an option that may contribute to an equitable and inclusive learning environment.

Paudel [11] stated that instructors and learners interacting with one another in a meaningful way indicates a successful virtual training experience. Interactive learning highlights the shift from instructor-controlled to self-directed (on the learners’ part) learning [11]. With the range and features of technology at the instructors’ disposal, virtual training can result in a more interactive experience [11]. The synergetic relationship between training and technology is ubiquitous and organizations must be afforded ongoing support and be able to appreciate and advance the opportunities provided by virtual training [12].

Given the current rapid transition toward wide-scale adoption of virtual training, it is crucial to provide high-quality training to enable health care providers to properly perform their clinical tasks. As a response to the COVID-19 pandemic, the Digital Education department at the University Health Network (UHN) has moved all training sessions to a virtual environment. Prior to the pandemic, Digital Education offered face-to-face sessions, e-learning, and blended learning. The instructor-led sessions are currently being taught through videoconferencing platforms that allow learners to attend sessions remotely. Although organizations have embraced technology-enabled learning during the pandemic, it is critical to ensure that the changes are sustainable after the pandemic. There is a dearth of in-depth and qualitative virtual training research in health care; therefore, this study was conducted to bridge this gap. The objective of this study was to examine instructors’ and learners’ perceptions regarding the effectiveness of virtual training amid the COVID-19 pandemic by determining the challenges and opportunities faced in the implementation of virtual training in the context of health care settings and health information systems.

## Methods

### Study Design

This qualitative study was guided by the Technology Acceptance Model, which was developed by Davis [13-15] to understand factors influencing the adoption and implementation of information technologies. The most proximal antecedent to the actual use of a technology is behavioral intention, which is affected by one’s attitude [16]. Attitude toward behavior is thus influenced by two determining factors: perceived ease of use and perceived usefulness. *Perceived ease of use* is described as the effort an end user must apply to successfully use the technology, whereas *perceived usefulness* is the extent to which the end user believes that the technology will help improve their performance at work [16]. This study was approved as a quality improvement project and was granted an exemption by the UHN Research Ethics Board (QI ID: 20-0100).

The Community of Inquiry framework is based on a collaborative-constructivist approach [17], where reflection, critical discourse, and sustained dialogue enable learners to make both personal meaning and collective knowledge [18]. The symbiotic relationship between social, cognitive, and teaching presence provides an environment that stimulates deeper thinking for learners, which further enhances their understanding of the content. Cognitive presence is a vital element in critical thinking that allows learners to gain knowledge through discussions [17]. However, the dearth of connection and collaboration in technology-mediated communication makes it challenging for learners to engage in a virtual learning community [17]. Social presence fosters a collaborative virtual learning environment by facilitating the process of building trust, developing respect, and critical thinking within the community of learners [18]. Teaching presence allows for higher levels of cognitive presence to be developed, by designing meaningful learning activities, encouraging individuals to be active learners, and modeling behavior [18]. Collaboration and critical thinking do not necessarily occur spontaneously, hence leadership is necessary to advance collaborative inquiry [18].

### Educational Intervention

UHN Digital Education offers virtual instructor-led training and e-learning to all incoming staff who will be using a health information system in their practice setting. The training was provided virtually using videoconferencing tools and led by an instructor with advanced health information system skills and adult education expertise. The training session was 4 hours in length, during which mini didactic lectures and demonstrations of the system were integrated with practice opportunities. The sessions were structured based on a few key objectives, which included a summary of the modules, navigation of the system, and an overview of frequently used features and functionalities. Learners then had the opportunity to practice and explore the various functional abilities of the system with respect to their roles.

### Study Participants

Given that the purpose was to identify major themes from a diverse range of perspectives, a maximum variation purposive sampling strategy was employed. Purposive sampling is a technique widely used in qualitative research to identify and recruit participants who are particularly knowledgeable or experienced with a phenomenon of interest [19]. It is also used to ensure that participants in interviews reflect the demographics of the larger study population [19]. This sampling approach requires that the research analyst identify criteria based on relevant diversity characteristics, in advance, then select participants who meet these criteria to obtain maximum variation in data [20]. Participants were recruited with email invitations sent by the research analyst. Recruitment continued until theoretical saturation was reached. Saturation was defined as the point after which the interviews would not yield any new themes. Individuals were eligible to participate if they were instructors or learners who had taken part in a virtual instructor-led session at UHN and were able to provide informed consent.

### Data Collection

One-on-one interviews were conducted virtually through Microsoft Teams platform (due to COVID-19 social distancing recommendations) and were on average 20 minutes in duration. The interviews were conducted by a research analyst (TJ) with experience in conducting qualitative interviews. The interviews were conducted until no significant new issues or ideas emerged and theoretical saturation was achieved. A semistructured interview guide was utilized to review participant experiences and suggestions for improving virtual instructor-led training within the organization. This format enabled the interviewer (TJ) to diverge and encourage participants to elaborate on their answers when necessary. The interview guide consisted of 6 open-ended questions for instructors and 4 open-ended questions for learners that explored the challenges and opportunities with the implementation of virtual training. The interview questions were modified based on an iterative process during data collection to challenge, refine, and elaborate on emerging themes. All participants provided informed consent for the interviews to be recorded; interview recordings were professionally transcribed verbatim.

### Data Analysis

An iterative, inductive, constant comparative process was used to thematically analyze the interview transcripts. The data were analyzed after the first 2 interviews, and the themes identified from that analysis were used to shape further data collection. Throughout the data collection and analysis process, a research analyst (TJ) and an education specialist (CH) with digital education backgrounds independently coded the data from an exploratory lens and generated a codebook. The data were first deductively analyzed using Technology Acceptance Model constructs as predefined codes. The data were then analyzed inductively, by following the systematic process outlined by Braun and Clarke [21]. Open coding was used when data did not adequately capture the predefined codes, and themes were inductively generated. Iterative biweekly discussions with content experts within our team (a manager of Digital Education and an education researcher) enabled us to further contextualize the themes. Qualitative data analysis software (NVivo, version 12; QSR International) was used to code and organize the data. Methodological rigor was achieved by assessing the quality of the thematic analysis using a 20-question evaluation tool [22]. An audit trail of each team member’s dependent coding, team meeting notes, and different versions of the coding structure were also maintained. Furthermore, sampling continued until theoretical saturation was achieved.

## Results

A total of 18 participants (female: 14/18, 78%; male: 4/18, 22%) agreed to participate in semistructured interviews. Of the 18 individuals who participated in the study, 9 were education specialists, 5 were learners, 3 were program coordinators, and 1 was a senior manager at the Centre for Learning, Innovation, and Simulation.

Three predominant themes were identified through a thematic analysis of the data, each with several subthemes: (1) adopting a learner-centered approach for a meaningful learning experience, (2) embracing the advances in educational technologies to maximize the transfer of learner, and (3) enhancing the virtual user experience.

### Theme 1: Adopting a Learner-Centered Approach for a Meaningful Learning Experience

#### Access to the Training Environment

Participants expressed that having access to a training environment would enable them to follow along during the training and provide an opportunity to practice afterwards. Participants commented on the importance of following along with the instructor as the instructor was demonstrating to enhance their learning experience (by following the complete functionality from beginning to end, rather than just parts of it).

…they have a lot of resources and have this amazing playground. It’s all web-based and people can just access it even offline without being monitored by the instructor. Just go in and play and click around learning the system. If we are able to provide that to UHN learners, that would be a huge advantage. And multiple people can go in at the same time, whereas at UHN our training environment, we kind of have to monitor it because we only have 10 logins. Well right now speaking of the current setup, like we only have 10 to 15 logins so only 15 people can log in at a time. Even then we kind of have to monitor, what if they change things around in the environment. We have to reset every evening.

ID 16

This is similar to a face-to-face instructor-led session, in which the instructor would be at the front demonstrating the functionalities as it is projected on the screen, and the learners would follow along at their own computer stations. Although currently, instructors work around this by granting screen control, learners feel that their learning experience may be affected slightly because only one person is able to use the training environment at a time.

#### Integration of Hands-on Experience and Interactivity

Nearly all participants desired hands-on practice as it allowed them to become familiarized with the system. They indicated that hands-on practice is very important for learning, since some individuals learn by experience.

Not everyone learns in the same way, where they watch and immediately understand how to do it. We need the hands-on, the muscle memory from experience. So that would be a huge advantage if we were able to provide that.

ID 16

A few participants described not being familiar with the new system; however, they were able to understand better when the instructor shared their screen and provided them with instructions on how to navigate and complete the tasks. The training provided them with the specific steps to critically reflect and attempt to understand not only the tasks themselves, but also the concepts underlying the tasks. Participants also reported that the interactive component of the session fostered collaboration between learners. For instance, the learners would help each other by saying what to click next when somebody was stuck as opposed to the instructors providing the answers.

It felt more intimate to then. It sounds weird, but I guess because everybody got a chance to do some stuff one on one, meaning everybody got a chance and everybody else got a chance to watch. I feel like people learned a lot more that way. I feel like people were more focused on learning as opposed to in class, they could pull out their phone, they could I mean, I guess they could still do that at home, too. But, you know, knowing that they had to prove their understanding by having to do examples in front of everyone else online. That then I feel like they paid more attention and they had good questions to ask.

ID 13

One participant stated that the physical action of practicing with the system enabled them to go through the process and effectively use it in their clinical environment. Other participants commented that it was more engaging than the classroom session since they knew they needed to take turns using the app and thus needed to understand what the person ahead of them did. With rapid advances in videoconferencing platforms, instructors felt that it was easier to use many classroom engagement techniques that would have been cumbersome to conduct in a live setting.

### Theme 2: Embracing the Advances in Educational Technologies to Maximize the Transfer of Learning

#### Awareness of Nonverbal Communication

Interestingly, awareness of nonverbal communication was identified by all instructors as a barrier to teaching virtually. Due to the lack of feedback and nonverbal cues, instructors felt it was challenging to assess engagement and knowledge comprehension in virtual learning. For example, in an in-person setting, if someone was not understanding a concept, the instructors could usually recognize this based on their facial expressions or how they were interacting with the keyboard.

You have to adapt it in a way that you can still convey your point sometimes without having to necessarily see, like actually see a person in front of you. So if I have some nonverbal cues and stuff when I'm speaking, you don't necessarily see that. With virtual classrooms, I did notice one of the challenging things was the lack of being able to see the participants of the class. It makes it kind of difficult to gauge if people are understanding or if I didn't repeat something like someone actually has to literally tell me or else I wouldn't be able to catch those nuances. Whereas if I was doing it in person, I can actually look at their face, their body language and see the cues and pick up the cues that way.

ID 7

Oftentimes, in virtual learning, they had to make assumptions about whether learners had absorbed the content presented and could successfully use the system in their clinical environment. They expressed that, sometimes, there are learners in the class who are quiet; however, this does not always necessarily mean they do not understand. This can be attributed to the fact that they may not know what to ask.

And being in the instructor's seat, you know, the silence is a lot harder, I find, to bear. Like, if you throw out a question to the group, right. And you do that live, you can sort of look around and catch eyes with somebody and say, like, OK, like lets somebody respond to this question because I'm not just going to drone on for an hour. I'm going to try to have you guys tell me something. But online, that's it seems like you're just kind of throwing it out into the void sometimes. And, yeah, it feels like it's just like the silences seem so much longer.

ID 10

#### Technical Limitations

Participants generally felt that a more stable platform for virtual delivery would have enhanced their teaching and learning experience. Most of the issues were technologically based (eg, some learners in the same session would be able to control the instructor’s screen while some could not). However, to mitigate this issue, instructors identified workarounds such as navigating the screen on behalf of the learners, with the learners providing instructions on how to approach the task. Technology was a barrier for many participants due to poor internet connection or not having access to a webcam or headset. Additionally, instructors stated that a major challenge was finding a balance while teaching virtually so that they were not only catering to one type of learner, but to all types of learners, while also managing the technology. Most of the participants contended that it would take time to become familiar with the platform and how it could be leveraged to run a virtual session in a fulsome way. Thus, it is vital to provide some scaffolding for those who are learning how to use the technology.

So that that I found a bit of a challenge to the ones that, you know, when I talked about clicking your mouse here, something as simple as that, when, where if for someone who was like medium savvy on the computer. Yeah versus someone who has no idea how to work a computer. So something as simple as clicking on your mouse required an explanation.

ID 11

### Theme 3: Enhancing the Virtual User Experience

#### Need for a Positive User Experience for both the Instructor and the Learner

Analysis revealed that some of the tools used to teach virtually were not sufficient to properly host or facilitate a virtual session. Participants suggested that providing the hardware and infrastructure would facilitate their teaching process, since they encountered technical issues with some of the platforms.

I will say, though, that we need the proper tools to do it, because also with my experience, the tools that we used, we've used so far have not been the greatest. So, I mean, I would say [Platform A] is very simple to use. I use [Platform A] a lot without any issue, really, to be honest. And then but [Platform B], which is kind of what is being pushed or was being pushed for us to use to teach was not very stable. It crashed a lot. And so I didn't use it, to be honest. I know we were…encouraged to use it. But I refused.

ID 13

One participant referred to digital tools as merely a vehicle for content and pedagogy. They reported that translating the same 2-hour in-person session to a virtual format would neither make learning more efficient nor lead to the best knowledge transfer. Participants suggested that some of the videoconferencing tools promoted by the organization may not be sufficient to captivate the attention of learners and increase engagement. For instance, a few participants stated that certain virtual platforms were better for collaboration, but they were not tools for teaching. Such tools are critical as a part of the training is based on synchronous learning with a broader longitudinal conversation and engagement. Certain platforms were preferred as they provided the tools for teaching, such as live synchronous communication features, polls, annotations, and 2 types of chats. Although some platforms have the features available for teaching, the organization did not support these platforms and encouraged the use of the supported platform.

#### Fostering a Social Nature of Learning

A virtual community of learners was identified as a critical element in enabling learning and facilitating the sharing of best practices. One of the participants opined that based on the education theory, learning is essentially a social activity, and a majority of learning occurs outside of the synchronous lecture or teaching session. Additionally, participants stated that there should be a place for learners to be able to come together and have their own private sessions to do group work, share resources, or even have an informal chat. Existing virtual platforms enable the facilitation of this interprofessional virtual community of learners.

But I think the pandemic has allowed everybody to jump into the same pool and to be kind of because the challenge I've always had with virtual communities of practice is that, you know, they were a bit of a niche thing before. And if people weren't really fluid with interacting online, they wouldn't bother. So you would have this fragmented community where you would have some people who were virtually connected and other people who weren’t. And it would be kind of a mess. So now, you know, zoom is a verb. So like, everybody is pretty savvy, like regardless of platform, like everybody is pretty savvy on getting connected. So I'm actually pretty excited for what this means for virtual communities of practice.

ID 10

The COVID-19 pandemic has accelerated the need to enable a virtual community of learners and recognize that learning is a social construct. Furthermore, since these platforms are hosted within the core infrastructure, the information shared will require some oversight to ensure shared learning information is accurate.

#### Implications of the Technology Acceptance Model Framework

The COVID-19 pandemic has compelled instructors and learners to embrace the virtual training experience and overcome any hurdles that arise during the virtual teaching-learning process. It is imperative to understand the factors that influence the adoption and implementation of virtual training to ensure virtual training is fully optimized (Table 1). The Technology Acceptance Model has allowed us to understand the instructors’ and learners’ perspectives about virtual training, specifically, current challenges and future opportunities for implementing virtual instructor-led training in health care organizations’ postpandemic conditions. Many participants perceived the training was useful as it improved their learning performance and provided information they needed to successfully use the health information system in their clinical environment. When users perceive that the virtual platform is useful in learning, they tend to be more motivated to learn and use it. Others suggested that providing access to the training environment enables learners to deliberately practice with the health information system and increase their confidence. Thus, when the technology is easy to use, the desire to continue using the digital tool increases. Nevertheless, participants encountered technical challenges, which impacted ease of use of the virtual platform.

**Table 1:** Technology Acceptance Model constructs associated with the 3 major themes identified in the thematic analysis

Construct	Findings	Quotations
Perceived Usefulness	Improved learning performance and the session was efficient Learners were able to successfully use the technology in their practice setting	One participant mentioned “I learn by doing it myself and the way they did it. I was still able to learn by myself. It just wasn’t on my computer. I was just moving the mouse on somebody else’s computer but it was still hands-on because they directed me through everything I needed to go through. And they kind of troubleshooted at the same time in the sense that they’ll give you heads up. People normally have trouble with this and this. And this is how you avoid it. And that’s what they did. I found that very helpful when I was on the job.” [ID 18]
Perceived ease of use	Access to the training environment provides an opportunity for participants to practice independently as opposed to using the instructor’s computer to have access to a training database Lack of feedback and awareness of nonverbal cues made it challenging for instructors to understand if learners were following along	One of the learners stated, “I just liked it because it was so easy and I don't know, like I had never done anything like this before.” [ID 14] “To me, I found the workshop pretty straightforward, especially with the use of software I was using as well. It was pretty easy to grasp. I just needed to know how to workaround it. How to navigate it is what we needed to know. It was pretty straightforward and easy to learn.” [ID 18] An instructor reported “So it's very different than doing it virtually been in person. I find that a bit challenging. Sometimes it feels like you're just talking to a wall because no one's answering any questions or they're not saying if you're asking if sometimes you would pause, like, I would pause and ask you, are there any questions. And then there's complete silence and you can't see anything.” [ID 7]
Attitude	Videoconferencing tools with a more user-friendly interface and accessible outlets were perceived as superior tools among several participants	—^a^
Behavioral Intention	Instructors refused to use certain platforms as they experienced technical issues and used platforms that were more resilient and easier to use	—

^a^A quotation is not provided.

## Discussion

### Principal Findings

Although there are many papers [23-30] about virtual education, little is known about the efficacy of virtual synchronous training to prepare health care professionals to use health information systems as a part of their professional practice. Many papers [1,8,11,12,31-34] focused on web-based teaching-learning in higher education. Given the relative paucity of literature focusing specifically on virtual training in health care organizations, this study sought to understand instructors’ and learners’ perceptions regarding the effectiveness of virtual training. Our study advances the findings from a recent study by Shala and colleagues [2] and provides insights on the challenges and opportunities associated with transforming face-to-face instruction to a virtual format. Three themes were conceptualized and related to adopting a learner-centered approach for a meaningful learning experience, embracing advances in educational technologies to maximizing the transfer of learning, and enhancing the virtual user experience. The findings are situated based on the community of inquiry framework, and opportunities for future research are summarized.

This study highlighted the importance of adopting a learner-centered approach by integrating hands-on practice as a part of the training and providing access to the training environment. Instructors supported cognitive presence by providing an opportunity for the learners to practice with the system as it enabled learners to follow the instruction step by step and receive appropriate feedback. Additionally, instructors delineated that this would provide a significant advantage to learners—by observing their peers demonstrate functions, they would become more motivated and invested in their learning. Cognitive presence is heightened by taking into consideration different learning preferences, one of which includes hands-on experience. Merrill [35] stated that learning is promoted when reflecting on prior experiences, demonstrating and applying skills, and integrating them into real-world problems. Edwards and colleagues [36] have outlined that, by providing an opportunity to practice with the system, active learning is fortified, thus increasing learners’ confidence. Merrill [35] asserted that learning is enhanced when coaching and continuous feedback are used to guide their learning process. Scaffolding enables learners to have considerable support; however, as learning progresses, it is important to gradually attenuate the amount of guidance and provide the learner with greater control [35]. Similar to our study, previous research identified that access to a training environment provides an opportunity for continuous interaction with the system, and consequently, learners reduce the cognitive effort associated with performing a task [37]. Additionally, the instructors incorporated interactive components as a part of the training session to foster collaboration with the learners, thereby enhancing the social presence element, which is in alignment with the best practices for teaching. Social presence provides a propitious environment where learning can be successfully created and sustained [18]. Raza [38] and Chen [31] reported that platforms with rich interactive functionalities are critical for fortifying learners’ sense of connectedness and creating an inclusive virtual learning space.

Despite the opportunities for virtual training to penetrate new areas and offer sustainable and effective learning solutions, there are challenges that may encumber the organizations’ efforts to shift skills-based training to a virtual platform. Our study identified several challenges, including technical limitations, different levels of digital literacy among learners, and a need for a positive user experience for both the instructor and the learner. Another major challenge in the study was the instructor’s decreased ability to recognize struggling learners so that they may be assisted promptly. Mishra and colleagues [8] corroborated this finding in a study that reinforced the importance of nonverbal communication. The authors [8] reported that instructors were unable to read the facial expressions and body language of learners; hence, it was difficult to adapt teaching patterns. Moreover, the virtual format made it challenging for instructors to determine whether participants were actively present during the session [8,32]. Our results suggest that awareness of nonverbal communication is essential in establishing teaching presence in a virtual environment. Consequently, instructors will be able to ensure that the learners are continuously engaged and facilitate the discussion in a meaningful way [39]. Teaching presence was enhanced when the instructor provided constructive feedback and actively facilitated tasks, thus allowing learners to critically explore and evaluate the information learned. Swan et al [39] reported that the capacity that various technologies have for transmitting nonverbal and vocal cues is critical in enhancing social presence. The literature highlights that the rapid shift to virtual training has further exacerbated the digital gap and cultural leap [1].

The findings in this study underlined the importance of developing a virtual community of learners to distill knowledge and facilitate the sharing of best practices while balancing learners’ cognitive load. Participants reported that a virtual community enables a safe environment for individuals to engage in learning through collaboration. Virtual instructor-led training provides new opportunities to make learning accessible to many learners and support them in developing their competencies, skills, and attitudes [33]. Garrison [18] argued that community and the social context of education are important to learning. He asserted that learning experiences must be prudently designed and socially supported for progressive intellectual development. Social constructivism emphasizes that learning is dependent on social interaction and context, where shared meaning is developed through discussion and negotiation [18]. The social constructivist approach highlights the significance of culture and environment on the learning process [40]. Knowledge is created from the interactions with peers and their environments, and meaningful learning ensues when learners are engaged in a discourse [40]. Education models based on the social constructivist approach emphasize the need for collaboration among learners and experts in the field [40].

Developing a virtual community of learners that is anchored in the 3 elements of educational experience will foster cognitive development through individual knowledge construction and collaborative discourse [41]. A virtual learning community should embrace the collaborative-constructivist notion, where the learning environment is cocreated, thus facilitating a critical dialogue within the digital space [41]. This enhances the key facets of social presence by providing critical feedback to members, respecting interpersonal relationships, and stimulating divergent thinking [41]. The Community of Inquiry framework subsumes a learner-centered approach, where learners are engaged in the exchange of knowledge, critical inquiry, self-evaluation, and deep learning [17]. Learners illustrate higher confidence levels, greater engagement, and stronger problem solving capabilities [41]. The virtual delivery design should foster transformative learning and strongly align with the Technology Acceptance Model and Community of Inquiry framework in order for the training to be effective, regardless of whether training is synchronous, asynchronous, or blended.

Several opportunities were identified in this study, including the adoption of a learner-centered approach for a meaningful learning experience and fostering a social nature of learning through the development of a virtual community of practice. It is imperative to integrate high interactivity and practice exercises throughout the sessions to reinforce specific knowledge and skills [36]. Thus, opportunities to practice with the system and the ability to reflect on the knowledge that is gained are critical in aiding knowledge translation [42]. However, interactions with members within a community of practice can go beyond the acquisition of knowledge, which contributes to better learner outcomes and meaningful use of health information systems [43]. The digital learning space allows participants to share their knowledge and engage in a collaborative discourse with colleagues and experts.

### Limitations

The findings of our study must be examined in the context of the following limitations. The study is limited to a single Canadian health care organization in a large urban area, and though we attempted to be as inclusive as possible during purposive sample, not all areas of the organization were reached. However, this study offers perspectives from education specialists and learners, specifically, about the current challenges and future opportunities faced in implementing virtual instructor-led training in health care organizations in postpandemic conditions. Recognizing that researchers’ positions and perspectives inevitably influence access to findings, we asserted research rigor and reflexivity through the triangulation of data from multiple perspectives and memo writing.

### Conclusions

The COVID-19 pandemic is rapidly transforming the landscape of the health care system, affecting not only the delivery of care, but also, how health care professionals will acquire new skills and be educated in the future. Training is vital in enabling care providers and staff to navigate the health information system safely and securely while providing optimal care. Virtual training has significantly reshaped how we teach and engage with our learners to maximize the teaching experience and lead to better patient care [23]. Organizations can build on this change to create immersive virtual learning spaces that can achieve intended educational outcomes while ensuring optimal learning experiences. It is incumbent to understand the key functionalities of the platform and the challenges associated with it for successful integration of digital technologies into virtual training [24]. The pandemic has challenged the health care system in unprecedented ways, and the need for innovative solutions to optimize educational endeavors has increased significantly. Nonetheless, it is imperative to ensure that these changes can be sustained in the long-term. As we move into the future, the impact of this emerging educational paradigm must be scrupulously evaluated and expanded.

## Abbreviations

UHN: University Health Network
